# The medication violations in racehorses at Louisiana racetracks from 2016 to 2020

**DOI:** 10.1002/vms3.724

**Published:** 2022-01-06

**Authors:** Pamela Waller, Izabela Lomnicka, Cam Lucas, Sara Johnson, Levent Dirikolu

**Affiliations:** ^1^ Equine Medication Surveillance Laboratory (EMSL) Department of Comparative Biomedical Sciences, School of Veterinary Medicine Louisiana State University Baton Rouge Louisiana USA

**Keywords:** AORC, ARCI, racehorses, racetracks

## Abstract

**Introduction/Background:**

The number of publications for most common drug violations in racehorses is limited. This study reports the most common medication violations in racehorses at four major racetracks in Louisiana between 2016 and 2020.

**Methods:**

During this 5‐year period, 27,237 blood samples and 25,672 urine samples collected during the course of normal race meeting activities were analysed by initial screening procedure utilizing Liquid Chromatography Mass Spectrometry (LC‐MS/MS). Following initial screening, suspect samples were subject to quantitative or semi‐ quantitative confirmation analysis by LC‐MS/MS.

**Results:**

The total number of violations reported was 534 (1.01% of the total number of specimens analysed). The total number of violations reported in Thoroughbred horses was 210 while the total number of violations reported in Quarter Horses was 324. The percentage of total violations was %0.59 for all the specimens analysed in Thoroughbred horses while this percentage was %1.9 for all the specimens analysed in Quarter Horses during this 5‐year period. The most frequent violations included the overages (concentrations of permitted medications equal to or exceeding the set threshold) of clenbuterol (165 violations), non‐steroidal anti‐inflammatory drugs (NSAIDs) such as phenylbutazone (73 violations), combination of phenylbutazone with flunixin (45 violations) and muscle relaxant methocarbamol (40 violations).

**Discussion/Conclusions:**

The total number of violations were relatively low during 5‐year period, but wide varieties of medications with different pharmacological actions were confirmed in performance horses in Louisiana. The most frequently reported violations in Louisiana were for permitted therapeutic medications (clenbuterol, phenylbutazone, flunixin methocarbamol) with established threshold and/or withdrawal guidelines in racehorses.

## Graphical abstract

This study reports the most common medication violations in racehorses at four major racetracks in Louisiana between 2016 and 2020. The most frequent violations included the overages (concentrations of permitted medications equal to or exceeding the set threshold) of clenbuterol (165 violations), non‐steroidal anti‐inflammatory drugs (NSAIDs) such as phenylbutazone (73 violations), combination of phenylbutazone with flunixin (45 violations), and muscle relaxant methocarbamol (40 violations).

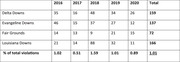

## 1 INTRODUCTION

The number of test samples for drug screening by anti‐doping laboratories continues to grow. Thus, the challenge faced by equine and human doping control laboratories is the increase in easily available drugs that are capable of affecting performance of equine and human athletes (Liu et al., 2011). Increased access to drugs that are now available without prescriptions through internet purchase further compounds the problem. Today, many drugs known to have a performance‐enhancing effect are prohibited in racehorses by the Association of Racing Commission International (ARCI). Within these guidelines, ARCI has established five classes of prohibited substances ranging from Class 1 substances, those demonstrating the greatest potential to enhance performance, to Class 5 substances, those demonstrating the least potential for enhancing performance ( Drug Testing Standards and Practices Program, 2020; ARCI, 2021a, b). Drugs that are intended for use in horses are found in lower classes. Drugs that are not intended for use in horses are placed in higher classes, particularly, if they might affect the outcome of a race. Drugs that are recognized as legitimately useful in racehorses but could affect the outcome of a race are found in the middle or higher classes (Drug Testing Standards and Practices Program, 2021; ARCI, 2021a, b)).

The ARCI Drug Classification Scheme is based on pharmacology, drug use patterns and the appropriateness of a drug for use in the racing horse. Drugs that are known to be potent stimulants or depressants are placed in higher classes, while those that have little effect on the outcome of a race are placed in lower classes. Some considerations are also given to placement of drugs based on practical experience with their use and the nature of positive tests. Twenty‐eight medications are currently approved for usage in performance horses with established thresholds and/or withdrawal guidelines by ARCI (2021b). Additionally, there are 11 permitted medications in racehorses with established thresholds with no withdrawal guidelines for endogenous, dietary or environmental substances by ARCI (2021a). All other drugs that are not regulated by ARCI with appropriate thresholds have zero tolerance rule (any level is considered violation). However, individual state Racing Jurisdictions can also develop their own racing rules and guidelines in addition to ARCI. For example, Louisiana State Racing Commission (LSRC) recognizes that certain ARCI Class 1 and 2 substances not natural to horses may be detected in trace amounts in official samples collected after race due solely to their prevalence in nature and/or the racing environment. For that reason, LSRC set threshold for methamphetamine regardless of isomer status (dextro‐ or levo‐methamphetamine) in urine at 10 ng/mL in 2020, and for cocaine, based on its major metabolite benzoylecgonine, in urine at 150 ng/mL in 2000. The analytical laboratories may function under differing rules and regulations in each jurisdiction, either on a country or on state basis. While most jurisdictions mandate analysis of samples for the broadest possible coverage of drugs, some analyse for a more restricted scope. Additionally, while certain medications are approved for use in racehorses in certain states, other states may have zero tolerance rule for such medications. For example, amino caproic acid, carbazochrome, ethacrynic acid, bumetanide, estrogen and ergonovine are listed as permitted adjunct bleeder medications in Louisiana. It is strongly recommended that racetrack veterinarians and trainers should be familiar with the rules and regulations of permitted medications in racehorses at various racing jurisdictions.

The number of publications for most common drug violations in racehorses is limited (Moss, 1984; Taddei et al., 2011). The goal of this manuscript was to provide information for the racetrack veterinarians, trainers and the public about the most common drug violations observed in racehorses using four major racetracks, Delta Downs, Evangeline Downs, Fair Grounds and Louisiana Downs, in Louisiana between 2016 and 2020.

## 2 MATERIALS AND METHODS

### 2.1 The laboratory and samples

Equine Medication Surveillance Laboratory (EMSL) is a second‐party laboratory within Comparative Biomedical Sciences (CBS) that falls under the parent company of Louisiana State University School of Veterinary Medicine, Baton Rouge, LA. The EMSL tests racehorses for prohibited substances for LSRC. Samples for testing arrived to the EMSL by courier delivery services. Samples were removed from packaging and verified against EMSL pre‐ or post‐race track shipping forms that come with the samples. All samples were received with a barcode placed by the LSRC. This study considered analysis of samples taken during the course of normal race meeting activities and did not require Ethics Committee approval.

### 2.2 Instrumentation and methodology

The analytical methodologies used for the plasma and urine screening were validated as per the ISO: IEC 17025:2017 guidelines for horse racing laboratories in compliance with Association of Official Racing Chemists (AORC,^2021^) and ARCI standards. The identification of substances was based on the compound's chromatographic and mass spectrometric properties. Instrument mass calibration (positive and negative), cleaning of sweep cone and ion transfer tube was done before every batch run. After calibration and cleaning, a system suitability test was performed before every batch run to verify the instrument performance and its suitability for the run. The methods used were validated on the following parameters including system suitability, specificity, identification capacity, matrix effect, precision, extraction efficiency, limit of detection (LOD) and carry‐over. Liquid chromatography high‐resolution mass spectroscopy screening analysis was performed using Thermo Fisher Q Executive UHMS Hybrid Quadrupole‐Orbitrap Mass Spectrometer (LC/MS‐MS). Chromatographic separation was achieved with an ACE Excel, 5 μm, C18, 2.1 × 75 mm column (ACT, Aberdeen, Scotland). Gradient elution started with 0.1% formic acid in water (98%) (Solvent A) and 0.1% formic acid in acetonitrile (2%) (Solvent B) at the flow rate of 0.3 mL/min. Every analytical batch was accompanied by quality‐control measures that included analysis of appropriate blank(s), positive and negative controls. The plasma screening procedure consisted of extraction using OASIS HLB 96‐well SPE plate and the urine screening procedure consisted of extraction using Biotage Isolute SLE 96‐well plate followed by identification and detection using Thermo Q‐Exactive high‐resolution mass spectrometer coupled to a LC.

After the screening procedure, if a sample was a suspect for any performance enhancing substance, various instrumentations in EMSL were utilized for quantitative/semi‐quantitative confirmation method using three Thermo Fisher Q‐Exactive Mass Spectrometer, one TSQ Vantage, one TSQ Access and one LTQ Velos LC‐MS/MS systems (Thermo Fisher, San Jose, CA) according to the EMSLs standard operating procedures. All the confirmation methods were performed using AORC Guidelines for the Minimum Criteria for Identification by Chromatography and Mass Spectrometry document (AORC, 2021).

## 3 RESULTS

During the 5‐year period (2016–2020), the laboratory analysed 52,909 samples (27,237 blood samples and 25,672 urine samples) collected post‐race from Thoroughbred and Quarter Horses from four racetracks in Louisiana (Table 1). Tables 2 and 3 summarize the total number of blood and urine samples analysed annually between 2016 and 2020 in Thoroughbred and Quarter Horses, respectively. The total number of violations reported was 534 (1.01% of the total number of specimens analysed) (Tables 4 and 5). The total number of violations reported in Thoroughbred horses was 210 while the total number of violations reported in Quarter Horses was 324 (Tables 4 and 5). The percentage total violations was 0.59% for all the specimens analyzed in Thoroughbred horses while this percentage was 1.9% for all the specimens analysed in Quarter Horses during this 5‐year period (Tables 4 and 5).

**TABLE 1:** Number of samples analysed by the EMSL from four major racetracks in Louisiana (2016‐2020)

	2016	2017	2018	2019	2020	Total
Blood	5867	5902	5846	5886	3736	27,237
Urine	5547	5630	5624	5422	3449	25,672
Total	11,414	11,532	11,470	11,308	7,185	52,909

**TABLE 2:** Number of Thoroughbred horse samples analysed by the EMSL from four major racetracks in Louisiana (2016‐2020)

	2016	2017	2018	2019	2020	Total
Blood	4028	4031	4040	3984	2531	18,614
Urine	3801	3841	3824	3658	2348	17,472
Total	7829	7872	7864	7642	4879	36,086

**TABLE 3:** Number of Quarter Horse samples analysed by the EMSL from four major racetracks in Louisiana (2016‐2020)

	2016	2017	2018	2019	2020	Total
Blood	1839	1871	1806	1902	1205	8623
Urine	1746	1789	1800	1764	1101	8200
Total	3585	3660	3606	3666	2306	16,823

**TABLE 4:** Number of violations and their ARCI classifications in Thoroughbred racehorses from four major racetracks in Louisiana (2016‐2020)

	2016	2017	2018	2019	2020	Total
ARCI Class 1	1	0	0	9	1	11
ARCI Class 2	1	1	3	11	8	24
ARCI Class 3	1	5	9	8	7	30
ARCI Class 4	58	25	25	17	13	138
ARCI Class 5	0	0	3	1	3	7
% of total violations	0.78	0.39	0.51	0.60	0.66	0.59%

**TABLE 5:** Number of violations and their ARCI classifications in Quarter racehorses from four major racetracks in Louisiana (2016‐2020)

	2016	2017	2018	2019	2020	Total
ARCI Class 1	0	1	0	3	2	6
ARCI Class 2	3	1	1	4	3	12
ARCI Class 3	13	6	124	34	8	185
ARCI Class 4	39	19	15	27	18	118
ARCI Class 5	0	0	2	0	1	3
% of total violations	1.54	0.74	3.94	1.85	1.39	1.9

In the study presented here, the most common violations were reported for ARCI Class 4 medications followed by Class 3, Class 2, Class 1 and Class 5 medications (Tables 4 and 5). During this 5‐year period, the proportion of the samples tested positive in Thoroughbred racehorses was highest in 2016 (0.78% of total samples) followed by 2020 (0.66%), 2019 (0.60%), 2018 (0.51%) and 2017 (0.39%) (Table 4). It is important to note that all of the racetracks in Louisiana were closed for approximately 2–3 months in 2020 with cancellation of both Thoroughbred and Quarter horseracing due to COVID‐19 pandemic. For Quarter Horses, the proportion of the samples tested positive was highest in 2018 (3.94%) followed by 2019 (1.85%), 2016 (1.54%), 2020 (1.39%) and 2017 (0.74%) (Table 5). .

The number of violations by racetracks per year in Louisiana are summarized in Table 6. Most of the medication violations were from Louisiana Downs racetrack (166 violations), followed by Delta Downs (159 violations), Evangeline Downs (137) and Fair Grounds (72 violations) racetracks. Interestingly, the number of clenbuterol violations reported in Louisiana racetracks was unusually high in 2018 in comparison to other years and there were total of 124 clenbuterol violations in 2018. Of these 124 clenbuterol violations, 65 violations came from Louisiana Downs, 38 violations from Delta Downs, 16 from Evangeline Downs and 5 from Fair Grounds racetracks. Almost all of the clenbuterol violations were reported in Quarter Horses (total 118 violations) with only six clenbuterol violations in Thoroughbreds in 2018.

**TABLE 6:** Number of violations by racetracks per year in Louisiana (2016‐2020)

	2016	2017	2018	2019	2020	Total
Delta Downs	35	16	48	34	26	159
Evangeline Downs	46	15	37	27	12	137
Fair Grounds	14	13	9	21	15	72
Louisiana Downs	21	14	88	32	11	166
% of total violations	1.02	0.51	1.59	1.01	0.89	1.01

The list of drug violations for 2016–2020 period is summarized in Table 7. The most common ARCI Class 1 drug reported was *d*‐methamphetamine (13 violations). Additionally, one violation was reported for each of the following ARCI Class 1 drugs: aminorex, Α‐pyrrolidinopropiophenone (α‐PVP), methadone and oxycodone. Levamisole was the most common ARCI Class 2 drug reported (18 violations) followed by mepivacaine (5 violations), caffeine (4 violations) and 3‐OH Lidocaine (3 violations). The most common ARCI Class 3 drug reported was clenbuterol (165 violations). Of these 165 clenbuterol violations, 150 violations were reported in Quarter Horses while only 15 violations were reported in Thoroughbred racehorses. Other common ARCI Class 3 drugs included albuterol (16 violations), detomidine (8 violations), acepromazine metabolite, 2‐(1‐hydroxyethyl) promazine sulphoxide (HEPS) (7 violations) and stanozolol (6 violations). In terms of ARCI Class 4 drugs, the most common violations were for phenylbutazone (73 violations), followed by phenylbutazone and flunixin combination (45 violations), methocarbamol (40 violations), triamcinolone acetonide (16 violations), flunixin (14 violations), combination of flunixin, phenylbutazone and ketoprofen (14 violations), and dexamethasone (13 violations). Over the 5‐year period, the number of reported violations with ARCI Class 5 drugs (Ranitidine and Omeprazole) was low on the list (10 violations) (Table 7).

**TABLE 7:** The violations reported in Thoroughbred and Quarter Horses from four major racetracks in Louisiana (2016‐2020)

Drug/substance names	Thoroughbred	Quarter Horse
**ARCI Class 1**		
Aminorex*	0	1
Α‐pyrrolidinopropiophenone (α‐PVP)	0	1
Methamphetamine (dextro)	10	3
Methadone	0	1
Oxycodone	1	0
Total	11	6
**ARCI Class 2**		
3‐OH Lidocaine^#^	0	3
Buprenorphine	1	0
Caffeine	1	3
Citalopram	2	0
Ketamine	1	0
Levamisole	15	3
Mepivacaine	4	1
Ractopamine	0	1
Tramadol	0	1
Total	24	12
**ARCI Class 3**		
2‐(1‐hydyroxyethyl) promazine sulfoxide (HEPS)^+^	7	0
Albuterol	3	13
Boldenone	0	4
Celecoxib	0	1
Clenbuterol	15	150
Detomidine	2	6
Guanabenz	1	0
Nefopam	0	1
Testosterone	2	1
Theophylline	0	1
Stanozolol	0	6
Xylazine	0	2
Total	30	185
**ARCI Class 4**		
5‐OH‐Dantrolene^X^	1	0
Ambroxol^!^	0	8
Betamethasone	1	0
Benzocaine	0	1
Dexamethasone	4	9
Dextrorphan^%^	7	1
Diclofenac	0	1
Dimethyl sulfoxide (DMSO)	0	1
Firocoxib	0	2
Flunixin	5	9
Isoflupredone	0	1
Ketoprofen	6	0
Methocarbamol	31	9
Methylprednisolone	0	7
Naproxen	1	0
Pentoxifylline	1	0
Prednisolone	1	0
Phenylbutazone	52	21
Phenylbutazone/Flunixin	17	28
Phenylbutazone/Flunixin/Ketoprofen	6	8
Triamcinolone Acetonide	5	11
Theobromine	0	1
Total	138	118
**ARCI Class 5**		
Ranitidine	3	1
Omeprazole Sulfide^&^	4	2
Total	7	3
TOTAL	210	324

* Metabolite of levamisole.

^#^ Metabolite of lidocaine.

^+^ Metabolite of acepromazine.

^X^ Metabolite of dantrolene.

^!^ Metabolite of bromhexine.

^%^ Metabolite of dextromethorphan.

^&^ Metabolite of omeprazole.

## 4 DISCUSSION AND CONCLUSIONS

The data obtained from four major racetracks in Louisiana indicated that ARCI Class 4 drugs were the most common violations (256 violations) in racehorses. This class was followed by ARCI Class 3 (215 violations), Class 2 (36 violations), Class 1 (17 violations) and then by Class 5 (10 violations) medications. ARCI Class 1 includes stimulant and depressant drugs that have the highest potential to affect performance and that have no generally accepted medical use in the racing horse. Many of these drugs are Drug Enforcement Agency schedule I and II substances. ARCI Class 2 includes drugs that have a high potential to affect performance. These drugs are not generally accepted as therapeutic agents in racing horses. Drugs in this class include psychotropic drugs, certain nervous system and cardiovascular system stimulants, depressants and neuromuscular blocking agents. Injectable local anesthetics are included in this class because of their high potential for abuse as nerve blocking agents. ARCI Class 3 includes drugs that may or may not have generally accepted medical use in the racing horse. Drugs in this class include bronchodilators, anabolic steroids and other drugs with primary effects on the autonomic nervous system, procaine, anti‐histamines with sedative properties and the high‐ceiling diuretics. ARCI Class 4 includes therapeutic medications that have less potential to affect performance than those in Class 3. Drugs in this class includes less potent diuretics, corticosteroids, NSAIDs, anti‐histamines, skeletal muscle relaxants without prominent central nervous system (CNS) effects, expectorants and mucolytics, haemostatics, cardiac glycosides and anti‐arrhythmics, topical anaesthetics, anti‐diarrheals and mild analgesics. Class 5 includes therapeutic medications that have very localized actions only, such as anti‐ulcer drugs, and certain anti‐allergic drugs.

The most frequent violations included the overages (concentrations of permitted medications equal to or exceeding the set threshold) of clenbuterol (165 violations), NSAIDs such as phenylbutazone (73 violations), combination of phenylbutazone with flunixin (45 violations) and muscle relaxant methocarbamol (40 violations). Because all these drugs are allowed medications in racehorses, there are thresholds established for clenbuterol, phenylbutazone, combination of phenlybutazone with flunixin, and methocarbamol, and therefore, their concentrations in blood and/or urine have to be monitored to differentiate acceptable therapeutic use versus administration for performance‐enhancing effects (Erichsen et al., 1994; Sasse and Hajer, 1978). The most common ARCI Class 1 and 2 drug violations were for *d*‐isomer of methamphetamine (13 violations) and levamisole (18 violations), respectively. The percentage total violations was 0.59% for all the specimens analysed in Thoroughbred horses while this percentage was 1.9% for all the specimens analysed in Quarter Horses during this 5‐year period.

Based on the findings of this study, the most common violation was reported for clenbuterol in racehorses, especially in Quarter Horses, in Louisiana. Prior to 2019, clenbuterol was a permitted medication in both Thoroughbreds and Quarter Horse racing with ARCI established thresholds and withdrawal guidelines by LSRC. The only approved formulation of clenbuterol used in racehorses is Ventipulmin syrup (Boehringer‐Ingelheim Vetmedica Inc., NADA 140‐ 973). Of 534 violations reported during 5‐year period in Louisiana, 165 violations were for clenbuterol. Some other racing jurisdictions also experienced unusually high number of clenbuterol violations. For example, New Mexico Racing Commission reported 71 violations in 2014, 84 violations in 2015, 57 violations in 2016, and 51 violations in 2017 for clenbuterol. Additionally, Florida Racing Commission reported over 125 clenbuterol violations in 2013. This unusually high number of clenbuterol violations especially in Quarter Horses resulted in banning of clenbuterol in Quarter Horses and other breeds racing with Quarter Horses (zero tolerance) in Louisiana by LSRC in 2019. Clenbuterol as Ventipulmin is used for the management of airway obstruction associated with respiratory disease in horses (Erichsen et al., 1994; Sasse and Hajer, 1978). While clenbuterol does provide bronchodilation, clenbuterol also has repartitioning effects on skeletal muscle that mimic the anabolic effects of androgenic/anabolic steroids (Dalrymple et al., 1984; Kearns et al., 2001, Reeds et al., 1986; Ricks et al., 1984; Spurlocket al., 2006). World Anti‐Doping Agency lists clenbuterol as a banned anabolic agent along with other beta‐2 agonists. Currently, ARCI Uniform Classification of Foreign Substances identifies clenbuterol as a Class 3 substance.

The second most frequent violations were for the overages of phenylbutazone (73 violations) and combination of phenylbutazone with flunixin (45 violations). A common practice in racetrack medicine in the USA is to administer the two NSAIDs within close proximity (24 hours apart) of each other. Phenylbutazone and flunixin meglumine are arguably two of the most commonly administered therapeutic substances in racetrack medicine. In equine medicine, phenylbutazone has continued to dominate the treatment of pain, particularly, that is associated with joint and muscle conditions (Tobin et al., 1986). Advantages of phenylbutazone in equine medicine include extensive clinical experience of efficacy and safety over both short‐ and long‐treatment periods, and availability in parenteral and a range of oral (powder, paste and bolus) formulations (Lees and Higgins, 1985; Lees et al., 1986). Flunixin is a NSAID analgesic and anti‐pyretic used in horses, cattle and pigs. It is often formulated as the meglumine salt (Lees and Higgins, 1985). Flunixin is recommended for the alleviation of inflammation and pain associated with musculoskeletal disorders in the horse. It is also recommended for the alleviation of visceral pain associated with colic in the horse (Knych et al., 2021; Ziegler et al., 2019). There are many FDA approved formulations of flunixin sold under different trade names (Banamine, Equileve, etc.) for use in horses. Currently, ARCI Uniform Classification of Foreign Substances identifies phenylbutazone and flunixin as Class 4 substances.

Methocarbamol is a permitted medication in racehorses with an established threshold and withdrawal guidelines by ARCI. Like phenylbutazone and flunixin, methocarbamol is classified as a Class 4 substance under the ARCI Uniform Classification of Foreign Substances. Methocarbamol is a centrally acting skeletal muscle relaxant labelled for use in horses as an adjunctive therapy for acute inflammatory and traumatic conditions of the skeletal muscle as well as to reduce muscular spasms (Knych et al., 2016). Injectable methocarbamol is FDA‐approved (Robaxin‐V; Fort Dodge Animal Health, Fort Dodge, Iowa) for use in horses for treatment of acute inflammatory and traumatic conditions of the skeletal muscle. Although oral methocarbamol is not FDA‐approved for use in horses, it is commonly administered orally to horses as 500‐mg tablets (as Robaxin‐V; Zoetis Inc.; Florham Park, NJ, USA) (Rumpler et al., 2014). Compounding pharmacies advertise that they prepare methocarbamol for use in horses in various formulations including oral powders, capsules, oral paste, oral suspension in oil and a transdermal gel.

The ARCI Uniform Classification of Foreign Substances currently identifies methamphetamine as a Class 1 substance. Methamphetamine is a strong CNS stimulant mainly used as a recreational drug and less commonly as a treatment for Attention Deficit Hyperactivity Disorder and obesity (Brewer et al., 2016; Cruickshank and Dyer, 2009; Knych et al., 2019). Methamphetamine exists as two enantiomers: dextro‐methamphetamine and levo‐methamphetamine (Brewer et al., 2016). Dextro‐methamphetamine is a much stronger central stimulant than levo‐methamphetamine (Brewer et al., 2016). There is no veterinary approved usage of methamphetamine in animals. l‐methamphetamine (absent any d stereoisomer) is present in over the counter inhalers with FDA approval for the treatment of cold and flu symptoms (vicks, vapour inhaler, etc.) (Brewer et al., 2016). There were 11 *d*‐methamphetamine violations in 2019 from four major racetracks in Louisiana. The LSRC recognizes that certain ARCI Class 1 and 2 substances not natural to the horse may be detected in trace amounts in official samples collected after race due solely to their prevalence in nature and/or the racing environment. For this reason, the LSRC established a urinary threshold for methamphetamine at 10 ng/mL regardless of the isomer found in a particular sample in 2020.

Levamisole is an anti‐helminthic drug and gained forensic interest after it was found that it was used as a cocaine adulterant. It also has conventional off‐label uses in horses as an immune stimulant and as a medication for treatment of Equine Protozoal Myelitis. Currently, ARCI Uniform Classification of Foreign Substances identifies levamisole as a Class 2 substance with no established threshold and/or withdrawal time in horses. Levamisole metabolizes in the horse to aminorex and possibly pemoline, both of which are potent stimulants and assigned a Class 1 Classification in the ARCI Uniform Classification of Foreign Substances (Drug Testing Standards and Practices Program, 2020; Gutierrez et al., 2010; Knych et al., 2019). Aminorex in horse urine is usually present as a metabolite of levamisole. However, a recent study reported identification of aminorex in horse urine with no history or evidence of levamisole administration. Analysis of the horse urine samples in this study suggested a botanical source directing attention to the Brassicaceae plant family as possible sources of aminorex (Maylin et al., 2019).

In conclusion, the most frequently reported violations in Louisiana were for permitted therapeutic medications (clenbuterol, phenylbutazone, flunixin, methocarbamol) with established threshold and/or withdrawal guidelines in racehorses. In this note, it is important to emphasize that the ARCI Controlled Therapeutic Medication Schedule for Horses list changes regularly. These changes include removal or addition of certain medications along with changes in recommended doses, thresholds, and withdrawal guidelines. Since the majority of the medication violations were for permitted medications with establish threshold and/or withdrawal guidelines, it is extremely important that the racetrack veterinarians and horse trainers are familiar with changes to the ARCI rules and regulations when it comes to using medications in racehorses. The risk of a therapeutic medication overage can be substantially reduced if the substances are administered at recommended doses. Conversely, large dose, long half‐life substances or formulations structured for prolonged pharmacological effect are at dramatically greater risk of producing an inadvertent therapeutic medication overage (Tobin et al., 2013). Given these circumstances, the optimal strategy is to select medications and administration routes resulting in the shortest detection times possible, and to make statistically appropriate allowance for the major unknown in this process, namely, horse‐to‐horse biological variability (Tobin et al., 2013). It is also important that the racetrack veterinarians and trainers should strictly follow the ARCI Controlled Therapeutic Medication Schedule for Horses in terms of dosing, route of administration, dosing interval and duration of treatment, in addition to, drug formulations approved when using therapeutic medications in racehorses to avoid medication violations. Additionally, the racetrack veterinarians and trainers should also be familiar with rules and regulations established by a Racing Commission in a particular state to avoid any medication violations.

## AUTHORS’ CONTRIBUTION

Izabela Lomnicka: Formal analysis; investigation; methodology; supervision; validation; writing—original draft; Writing—review & editing. Cam H. Lucas: Formal analysis; investigation; methodology; supervision; validation; writing—review & editing. Sara Johnson: Formal analysis; investigation; methodology; writing—original draft; writing —review & editing. Levent Dirikolu: Investigation; methodology; project administration; supervision; validation; writing—original draft; writing—review & editing.

## CONFLICT OF INTEREST

The authors declare no conflict of interest.

## ETHICS STATEMENT

The authors confirm that the ethical policies of the journal, as noted on the journal's author guidelines page, have been adhered to. This study considered analysis of samples taken during the course of normal race meeting activities and did not require Ethics Committee approval.

### PEER REVIEW

The peer review history for this article is available at https://publons.com/publon/10.1002/vms3.724

## Data Availability

The data that support the findings of this study are available from the corresponding author upon reasonable request.
